# Morpho-Physiological Classification of Italian Tomato Cultivars (*Solanum lycopersicum* L.) According to Drought Tolerance during Vegetative and Reproductive Growth

**DOI:** 10.3390/plants10091826

**Published:** 2021-09-02

**Authors:** Veronica Conti, Marco Romi, Sara Parri, Iris Aloisi, Giovanni Marino, Giampiero Cai, Claudio Cantini

**Affiliations:** 1 Department of Life Sciences, University of Siena, 53100 Siena, Italy; marco.romi@unisi.it (M.R.); sara.parri@student.unisi.it (S.P.); giampiero.cai@unisi.it (G.C.); 2 Department of Biological, Geological and Environmental Sciences, University of Bologna, Via Irnerio, 40126 Bologna, Italy; iris.aloisi2@unibo.it; 3 National Research Council of Italy, Institute of Sustainable Plant Protection (CNR-IPSP), Via Madonna del Piano 10, 50019 Sesto Fiorentino, Italy; giovanni.marino@ipsp.cnr.it; 4 National Research Council of Italy, Institute for Bioeconomy (CNR-IBE), 58022 Follonica, Italy; claudio.cantini@ibe.cnr.it

**Keywords:** local cultivars, stress tolerance, physiology, agrobiodiversity

## Abstract

Irrigation is fundamental for agriculture but, as climate change becomes more persistent, there is a need to conserve water and use it more efficiently. It is therefore crucial to identify cultivars that can tolerate drought. For economically relevant crops, such as tomatoes, this purpose takes on an even more incisive role and local agrobiodiversity is a large genetic reservoir of promising cultivars. In this study, nine local Italian cultivars of tomatoes plus four widely used commercial cultivars were considered. These experienced about 20 d of drought, either at vegetative or reproductive phase. Various physio-morphological parameters were monitored, such as stomatal conductance (g_s_), photosynthesis (A), water use efficiency (WUE), growth (GI) and soil water content (SWC). The different responses and behaviors allowed to divide the cultivars into three groups: tolerant, susceptible, and intermediate. The classification was also confirmed by a principal component analysis (PCA). The study, in addition to deepening the knowledge of local Italian tomato cultivars, reveals how some cultivars perform better under stress condition than commercial ones. Moreover, the different behavior depends on the genotype and on the growth phase of plants. In fact, the Perina cultivar is the most tolerant during vegetative growth while the Quarantino cultivar is mostly tolerant at reproductive stage. The results suggest that selection of cultivars could lead to a more sustainable agriculture and less wasteful irrigation plans.

## 1. Introduction

Water deficit is one of the major challenges of the 21st century and agriculture is both the cause and the victim because the 70% of global available water is used for agricultural practices [1]. Since water is fundamental for the life of plants in all the physiological processes [2] of plants, drought triggers a multitude of different responses affecting morphological and molecular traits in each phenological phase of plant growth [3]. Plants have evolved various adaptation mechanisms to counteract water scarcity, one of the most important is the stomata movements. Among these, one of the most important is the closure of stomata. When roots perceive water shortage, plants respond by increasing the synthesis of abscisic acid (ABA) [4], which leads to stomata closure [5]. Although stomatal conductance is then partially affected, a slight decrease in conductance has a protective effect against stress allowing plants to safeguard water reservoirs and improve water use efficiency [4]. In addition, morphological adaptations, such as stomatal density and leaf area, are involved in maintaining the water balance because a decrease of stomata number [6,7] as well as of the transpiring leaf surface [8] contribute significantly to reducing water loss. Defenses are not without side effects; when stomata close excessively, plants were compelled to activate scavenging systems, such as the water-water cycle, that counteract excess Reactive Oxygen Species (ROS) [9]. In addition, the synthesis of carotenoids allows to capture excess energy from chlorophylls and dissipate it as heat [9] although under extended stress conditions this process is not sufficient [10]. 

Drought also affects mitosis and consequently plant development reducing both cell number and expansion [11]. These events lead to a reduction in plant growth and yield lowering the revenues of the crop. For all these reasons, the selection of plants tolerant to water deficit has become a high priority. Locally adapted cultivars are the result of a domestication process of wild species that underwent selective pressures due to both contingent environmental conditions and human needs [12]. In addition, local cultivars are adapted to the various climate changes that a given environment may experience and, therefore, they show traits resilient to changing climatic conditions [13]. In this context, maintenance and protection of local agrobiodiversity become a resource for food availability [14]. Many studies identify local cultivars as a heritage of genetic traits that can make plants more tolerant to abiotic stress, such as drought [13,15,16,17,18,19,20]. For example, in countries like Peru, Brazil and India, recent repatriations of gene bank accessions raise questions about whether and how crop biodiversity can be included in production systems of areas prone to climate change [21]. In addition, agrobiodiversity is one of the global keystones in farming to secure stable harvest and livelihood under changing environmental conditions [22]. The greater is the supply of genetic diversity the greater the opportunities for farmers to adapt the crops to local environmental conditions. In this context, the access to a wide range of locally adapted cultivars is and will be pivotal for successive, sustainable agriculture under climate changes and extremes [23].

Tomato (*Solanum lycopersicum* L.) is one of the most important vegetable crops in the world, second only to potato [24]. Globally, almost 5 million of hectares of cultivated land is used for its cultivation, with a total of over 180 million of tons harvested fruits [25]. To date, Italy is among the top 10 tomato producers in the world with 5.2 million tons per year [25]. Tomato is particularly susceptible to water shortage because prolonged water deficit limits growth and yield of the harvest. Both vegetative and reproductive stages of modern tomato cultivars can be severely affected by drought, which inhibits seed development, reduces stem and fruit growth [26,27]. 

Previously, we evaluated the drought resilience of plants of seven tomato cultivars locally grown in the Tuscan territory (Italy) [28]. In that study, plants were cultivated in a growth chamber and analyzed for some key characteristics related to water deficit stress. In the present manuscript, the study was extended to all the nine tomato cultivars that are catalogued in the Regional Germplasm Bank of Tuscany as at risk of genetic erosion. Plants were analyzed for physiological (stomatal conductance, photosynthetic efficiency, water use efficiency, leaf relative water content) and morphological parameters (growth index, stem diameter, leaf area, stomatal density) as well as for soil water content. Plants were grown in greenhouse and analyzed either at vegetative or reproductive phases. The goal was to highlight the differences in drought tolerance that each cultivar might exhibit specifically in relation to either developmental stage [27,29]. Therefore, this study aimed to identify the most drought tolerant cultivars for future breeding to reduce irrigation demands in sustainable agriculture.

## 2. Results

### 2.1. Vegetative Phase

#### 2.1.1. Drought Stress Highlights Differences among Tomato Cultivars

**Figure 1:**
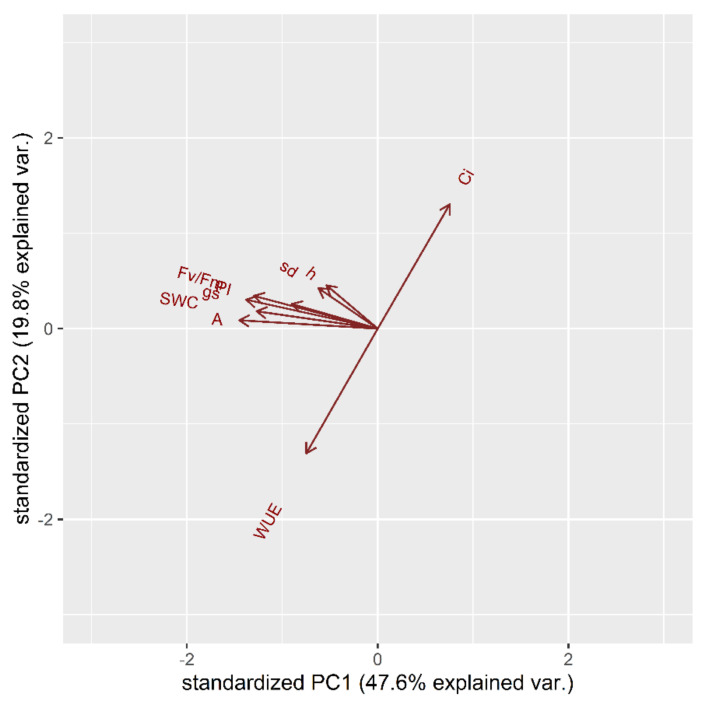
Principal component analysis (PCA) for physiological and morphological traits in the stress treatment at the vegetative stage: Water Use Efficiency (WUE), intercellular concentration of CO_2_ (Ci), photosynthesis (A), stomatal conductance (g_s_), Soil Water Content (SWC), photosynthetic efficiency (Fv/Fm), Performance Index (PI), height (h), stem diameter (sd).

At first, the multiple physiological traits were measured to perform a principal component analysis (PCA). The complete set of measured parameters at t_1_ was integrated to depict the correlation between the various traits. The time-point t_1_ (middle of stress treatment) was considered instead of t_2_ (end of stress treatment) since the latter was not determined by a varietal difference. Indeed, at t_2_ all the cultivars indiscriminately showed a too high deficit in most of the parameters examined. The first factor (PC1), to which the parameters A, g_s_, SWC and Fv/Fm contribute most, explains 47.65% of the total variance, while the second factor (PC2), to which WUE and Ci contribute most, about 19.8%. In total, both PCs explain 67.45% of the total variance of all analyzed variables. Figure 1 indicates that photosynthesis (A), conductance (g_s_), soil water content (SWC) and photosynthetic efficiency (Fv/Fm and PI) share a positive correlation. The height of plants (h) and the diameter of stem (sd) has a correlation between photosynthetic efficiency and Ci. The water use efficiency (WUE) is inversely correlated to the intercellular concentration of CO_2_ (Ci). 

**Figure 2:**
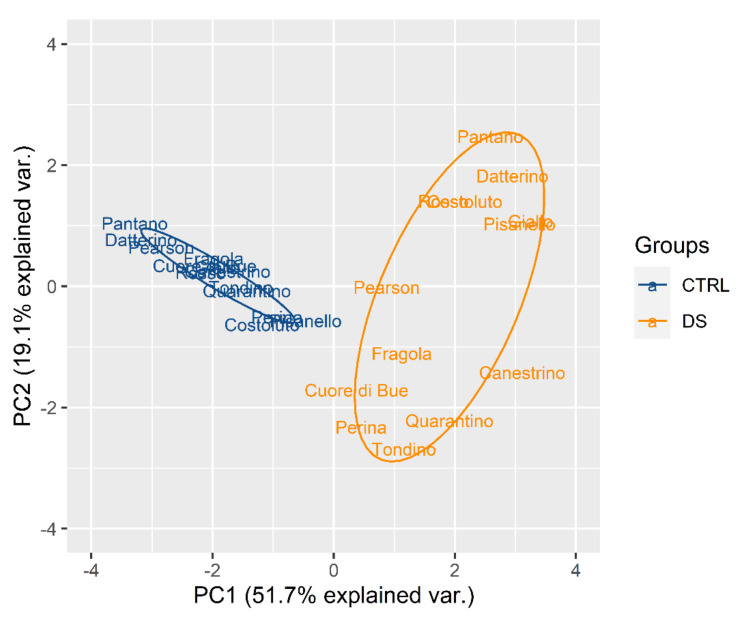
Principal component analysis (PCA) for genotypes based on control (blue) and stress (orange) indices calculated for physiological traits at t_1_ in the stress treatment at the vegetative stage.

In Figure 2 it is possible to notice that all control plants (blue) are distributed in a restricted area without much difference between the cultivars. On the contrary, all the stressed plants (orange) are distributed in a much larger space that extends mostly along the PC2 axis. This indicates that drought stress differentiates the behavior of plants in a genotype-dependent manner. It is important to observe that WUE and, correspondingly, Ci are the parameters that drive the differentiation between the genotypes.

**Figure 3:**
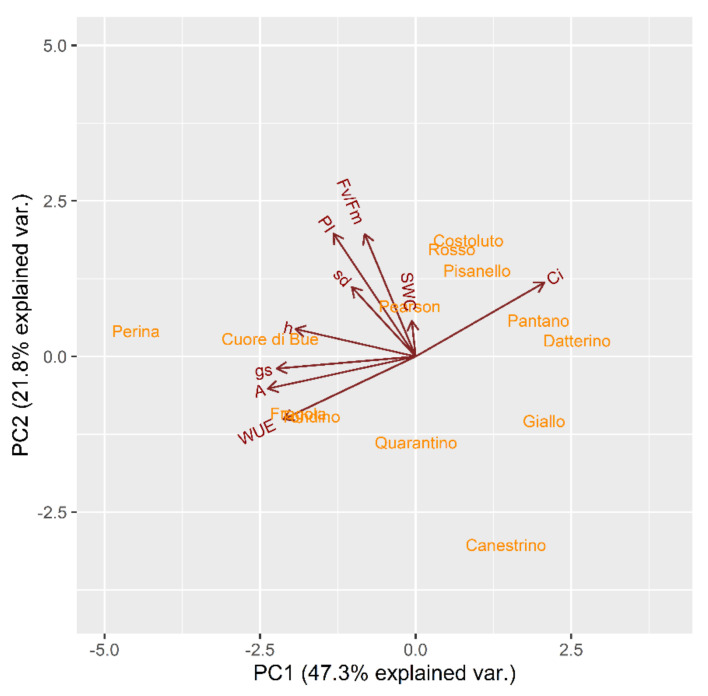
Principal component analysis (PCA) for genotypes based on stress indices in relation to control indices calculated for physiological traits at t_1_ in the stress treatment at the vegetative stage.

Secondly, to evaluate the behavior of each cultivar a PCA was performed with each parameter of the stressed plants in relation to their own control (Figure 3). Tomato cultivars can be divided into two main groups mainly by differences in PC1 values, which accounts for 47.3% of variation with high loadings of Ci, WUE, A, and g_s_. One group consists of Costoluto Fiorentino, Rosso di Pitigliano, Pisanello, Pantano, Datterino, Pearson, Giallo di Pitigliano and Canestrino di Lucca; the other group contains Perina, Cuore di bue, Fragola, Tondino. The genotypes of Quarantino and Pearson are at an intermediate position.

#### 2.1.2. Clusterization

**Figure 4:**
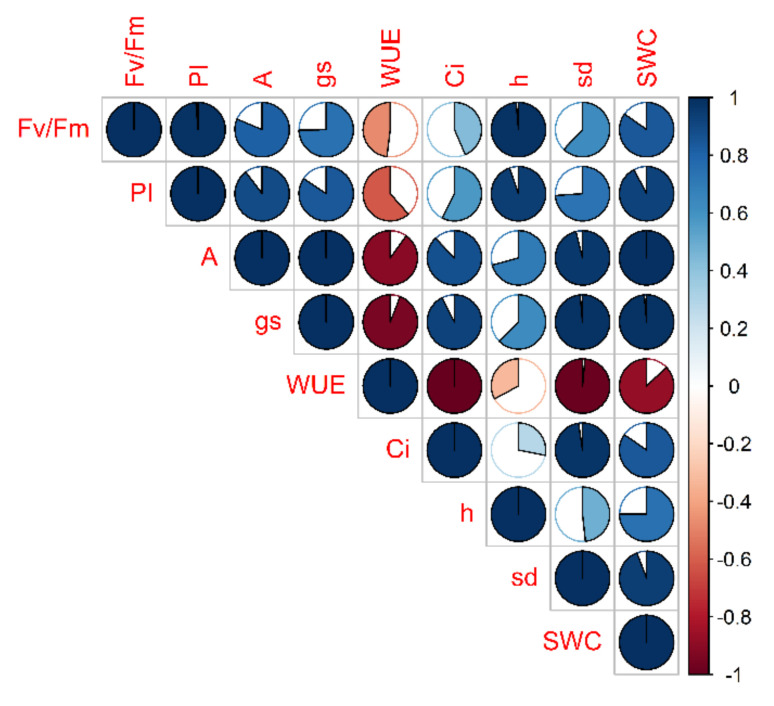
Correlogram of 9 physiological and morphological traits evaluated in Perina cultivar in the stress treatment at the vegetative stage. Each trait of DS plants is normalized to that of CTRL and then correlated according to time course (t_0_, t_1_, t_2_). The filling of the cake corresponds to the value of the correlation coefficient (full cake means unit correlation, in absolute value) while the color indicates the sign (blue/red means positive/negative correlation coefficient).

Nine traits have been correlated for each cultivar according to their time course. Firstly, each parameter relative to stressed plants was normalized to its own control (in percentage). Then, a correlogram for each cultivar was constructed (for a representative example see Figure 4). 

**Figure 5:**
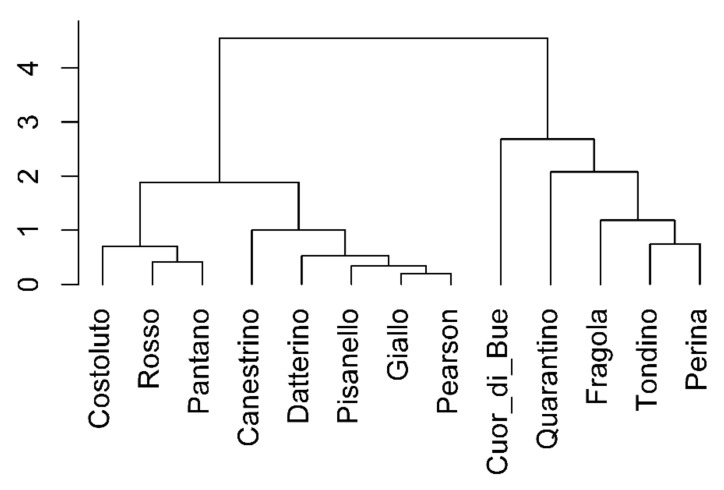
Dendrogram assembled by multivariate cluster analysis using correlation coefficients of all parameters with respect to WUE in the stress treatment at the vegetative stage.

From the PCA previously described, WUE and Ci turned out to be the parameters that most influenced the differentiation between cultivars. Hence, the correlations of all the traits with respect to WUE were used to construct the dendrogram in Figure 5 showing the cultivars distributed within two main clusters. One of them is clearly distinguishable and is formed by Cuore di bue, Quarantino, Fragola, Tondino and Perina. The other is composed by Costoluto, Rosso, Pantano, Canestrino, Datterino, Pisanello, Giallo, Pearson. A dendrogram corresponding to correlations with respect to Ci was also obtained, but it was not reported in this article as it revealed the same two distinct groups.

#### 2.1.3. Susceptible and Tolerant Cultivars

**Figure 6:**
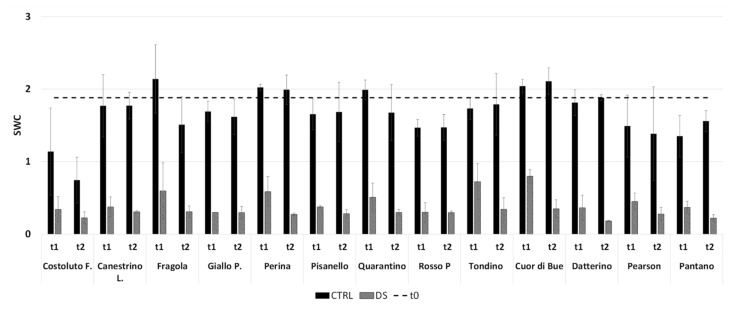
Soil Water Content (SWC) at t_1_ and t_2_ in the stress treatment at the vegetative stage. The dashed line indicates the initial SWC, at t_0_. In black are the controls (CTRL), while in stripes the stressed (DS). Vertical bars represent standard deviation of means of the values taken on 5 plants.

Analysis of clusterization and PCA revealed two very similar groups. Differentiation in these two groups can be encompassed by individual parameters. Perina, Fragola, and the commercial Cuor di Bue cultivars still have g_s_ quite far from 0 at t_1_. While Tondino, Quarantino, Costoluto and the commercial Pearson cultivars still have g_s_ near to but different from 0 at t_1_ (Figure S1). On the contrary, the remaining cultivars had a value already equal to 0 at t_1_. This allowed to find a first difference in perceiving water shortage as stress. As suggested by Galmes [30], it is valuable to observe the stomatal conductance together with the SWC. A non-vanishing value of g_s_ at t_1_ corresponds to SWC higher than 0.5 in the same cultivars (Figure 6), probably indicating that water is still available. Therefore, the different perception of water shortage as stress likely corresponds to a better management of the soil water resource in Perina, Fragola, Tondino, Quarantino and the commercial Cuor di Bue cultivars.

The literature reported that photosynthesis is one of the primary physiological targets of water stress [4,30,31]. Considering the values obtained from photosynthesis, Tondino Liscio, Quarantino, Fragola, Perina and Cuor di Bue again have A different from 0 at t_1_ (Figure S2). The parameter A can then provide an indication of the most tolerant genotypes.

WUE expresses the ability of a plant to produce biomass through photosynthesis per water consumed [30] and is considered a parameter useful for evaluating the best performing plants in conditions of drought stress [32]. In this study the most promising cultivars are Tondino Liscio, Quarantino, Fragola, Perina and Cuor di Bue (Figure S3), that can be considered tolerant to drought stress, while all the other cultivars are more susceptible to lack of water.

**Figure 7:**
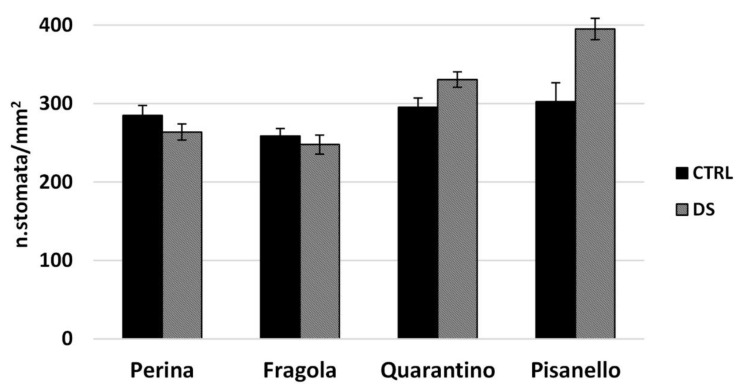
Stomatal density at t_2_, in the 4 representative cultivars in the stress treatment at the vegetative stage. In black are the controls (CTRL) and in stripes the stressed (DS). Vertical bars represent standard deviation of averages of the values taken on 10 photos for each leaf (three per plant).

Among all the cultivars, only four were selected for the next analyses. Combining all the results described so far, Perina and Fragola were chosen as representative of the group of tolerant cultivars. On the contrary, Pisanello was selected to be the most representative of susceptible traits among the local cultivars. Quarantino was selected as the medium cultivar that has both tolerant and susceptible characteristics. First, the stomatal density at t_2_ was calculated. As observed in Figure 7, the DS of Pisanello shows a higher and significantly different density compared to the CTRL, thus confirming a higher sensitivity to the stress [33]. The opposite happens to Perina, which has a lower density in the DS and significantly different from the CTRL, as to indicate an adaptation to drought stress. This result partly justifies the trend of WUE: a lower transpiration allowed a prolonged increase in the Perina compared to t_0_, while the increase in stomatal density may have affected the fall of WUE in the Pisanello cultivar. For Quarantino and Fragola the density is almost unchanged between CTRL and DS, indicating a non-susceptibility to stress of this parameter.

**Figure 8:**
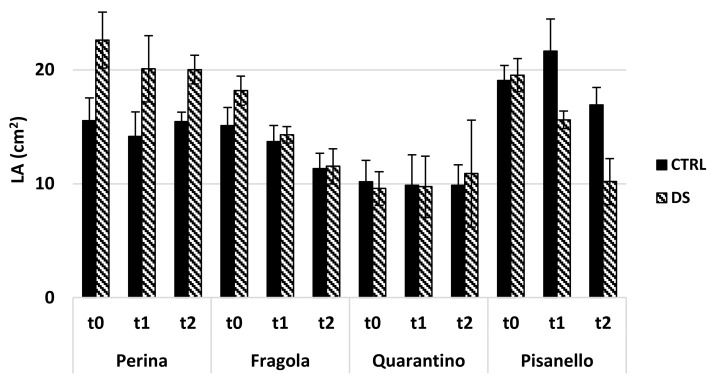
Leaf area (LA) of the 4 representative cultivars in the stress treatment at the vegetative stage. In black are the controls (CTRL) and in stripes the stressed one (DS). Vertical bars represent standard deviation of averages of the values taken on 3 leaves per plant.

The size of leaves plays a key role in the energy and water balance of plants [34,35,36] as a transpiring and photosynthesizing surface. The leaf area (LA) for the four cultivars at t_0_, t_1_ and t_2_ is shown in Figure 8. The stability of LA in Perina during the stress, together with the low stomatal density, confirms its excellent tolerance because it kept the photosynthesizing surface intact while it reduces transpiration. The LA of the DS of Quarantino and Fragola cultivars is also stable while that of Pisanello significantly decreases, differing significantly from the CTRL at t_1_. The damage was clearly visible as wilting and yellowing of plants. This confirms a strong sensitivity of Pisanello to drought stress.

### 2.2. Reproductive Phase

#### 2.2.1. Drought Stress Highlights Differences among Tomato Cultivars

**Figure 9:**
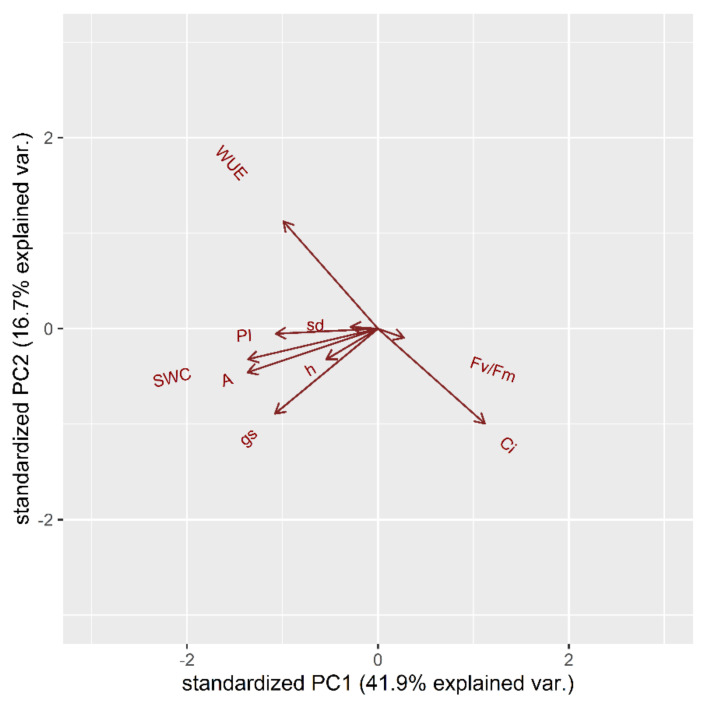
Principal component analysis (PCA) for physiological and morphological traits in the stress treatment at the reproductive stage: Water Use Efficiency (WUE), intercellular concentration of CO_2_ (Ci), photosynthesis (A), stomatal conductance (g_s_), Soil Water Content (SWC), photosynthetic efficiency (Fv/Fm), Performance Index (PI), height (h), stem diameter (sd).

As done for the vegetative phase, also in the reproductive phase a PCA was carried out with the multiple physiological data collected. The complete set of parameters at t_2_ was integrated to depict the correlation between the various traits. Photosynthesis (A), conductance (g_s_) and soil water content (SWC) have a positive correlation (Figure 9). There is a similar positive correlation also with water use efficiency (WUE) that is inversely correlated to intercellular concentration of CO_2_. The plants’ height (h) and the stem’s diameter do not show a positive correlation and the same occurs for Fv/Fm and PI. The first factor (PC1), to which A and SWC contribute most, explains 49.5% of the total variance, while the second factor (PC2), to which WUE and Ci contribute most, describes about 16.1% of total variance. Altogether, both PCs explain 65.6% of the total variance for all analyzed variables. 

**Figure 10:**
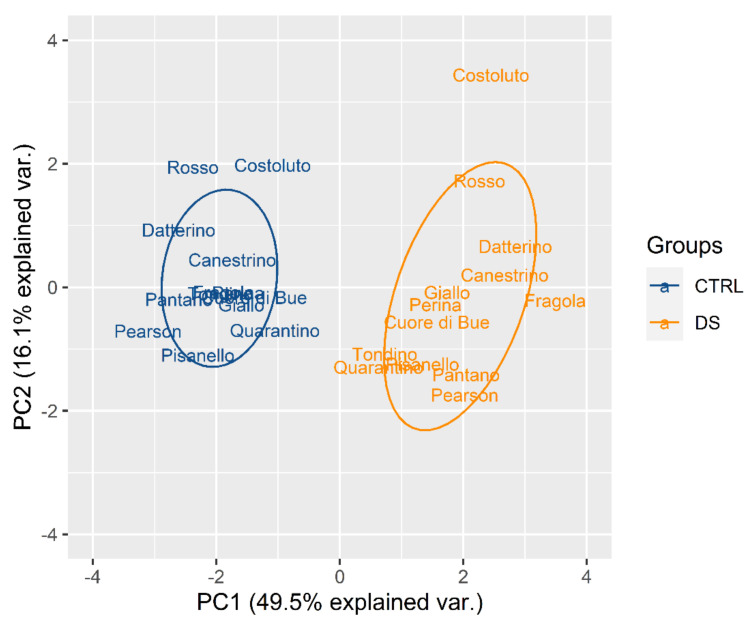
Principal component analysis (PCA) for genotypes based on control (blue) and stress (orange) indices calculated for physiological traits at t_2_ in the stress treatment at the reproductive stage.

Additionally, it was possible to clearly distinguish the control plants (blue) from the stressed ones (orange) (Figure 10). However, in the reproductive phase both control and stressed plants are distributed in a relatively large area, with some differences between the cultivars. This indicates that each cultivar has its own physiological behavior at the adult stage. However, drought stress indeed plays again an important role since the differentiation is more accentuated in the stressed (orange) group.

**Figure 11:**
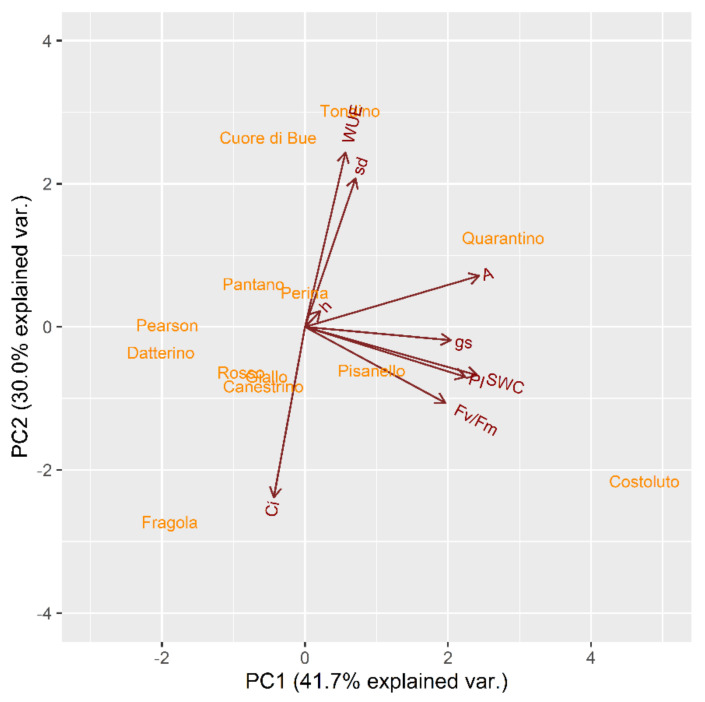
Principal component analysis (PCA) for genotypes based on stress indices in relation to control indices calculated for physiological traits at t_2_ in the stress treatment at the reproductive stage.

Secondly, to evaluate the behavior of each cultivar, another PCA was performed with each parameter of the stressed plants in relation to their own control (Figure 11). Following the same subdivision principle used for the vegetative phase, tomato cultivars can be divided into two main groups according to positive or negative values of PC1. In this case, one group consists of Costoluto Fiorentino, Pisanello, Tondino and Quarantino; the other group contains Fragola, Canestrino di Lucca, Giallo di Pitigliano, Rosso di Pitigliano, Datterino, Pearson, Pantano and Cuore di bue. The genotype of Perina is at an intermediate position.

#### 2.2.2. Clusterization

**Figure 12:**
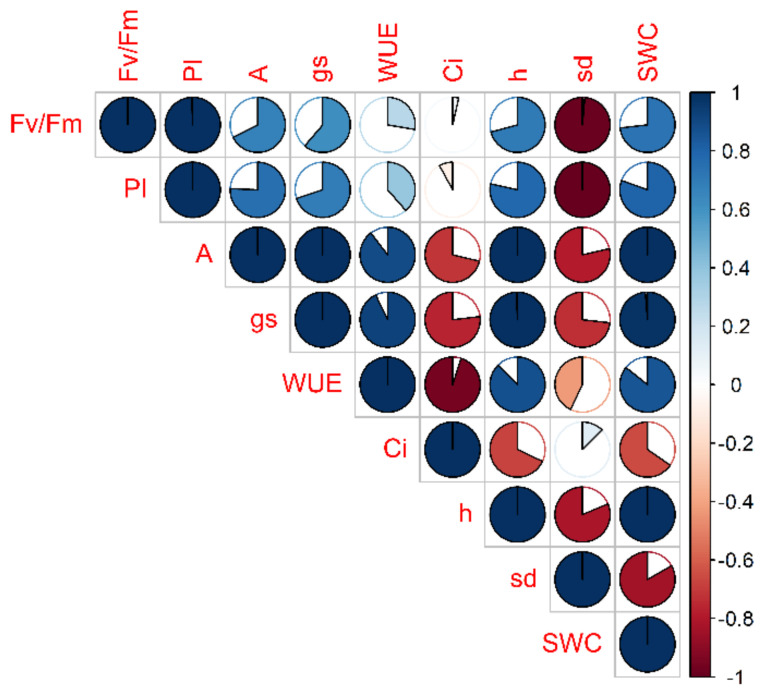
Correlogram of 9 physiologic and morphologic traits evaluated in the Perina cultivar during stress treatment atthe reproductive stage. Each trait of DS plants is normalized to that of CTRL and then correlated according to time course (t_0_, t_1_, t_2_). The filling of the cake corresponds to the value of the correlation coefficient (full cake means unit correlation, in absolute value) while the color indicates the sign (blue/red means positive/negative correlation coefficient).

**Figure 13:**
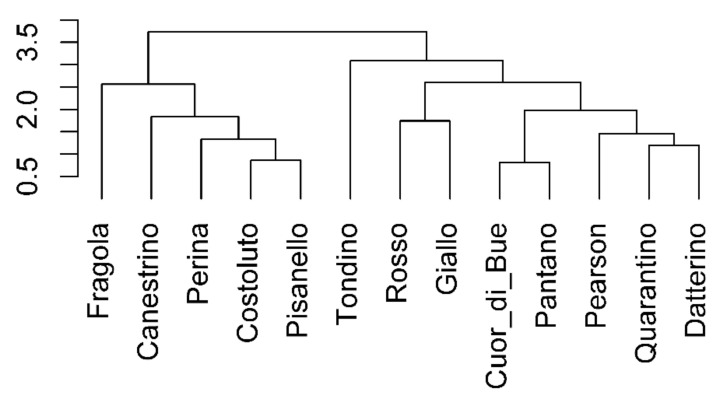
Dendrogram built by multivariate cluster analysis using correlation coefficients of all parameters with respect to WUE in the stress treatment at the reproductive stage.

A correlogram for each cultivar was constructed (Figure 12) on the base of nine traits according to their time course. The values related to stressed plants were normalized to their own control (in percentage). Following what done for the vegetative phase, the correlations of all the traits with respect to WUE were used to construct the dendrogram (Figure 13). In the reproductive phase two groups (clusters) are visible, but, with respect to the vegetative phase, groups are not too different. One is formed by Fragola, Canestrino di Lucca, Perina, Costoluto Fiorentino and Pisanello; the other is composed of Tondino, Rosso di Pitigliano, Giallo di Pitigliano, Cuor di bue, Pantano, Pearson, Quarantino and Datterino. 

#### 2.2.3. Susceptible and Tolerant Cultivars

The analysis of each individual parameter helps to understand the characteristics of cultivars and the differentiation between groups. Regarding stomatal conductance, the Quarantino cultivar has a g_s_ equal to 0.12 mol m^−2^ s^−1^, which is near to the value of its own control at t_2_ (Figure S4). The Perina, Giallo, Fragola, Canestrino, Rosso and the commercial Datterino, Pearson and Cuor di Bue cultivars have a g_s_ close to 0 at t_2_; in the commercial cultivars, the value of stressed differs greatly from their own control. The remaining cultivars have intermediate values between 0.06 and 0.09 mol m^−2^ s^−1^.

**Figure 14:**
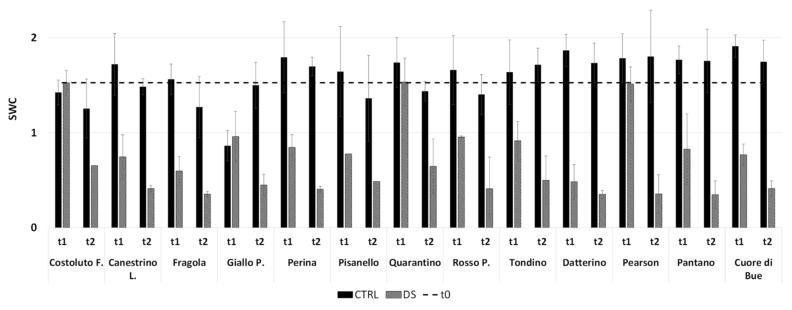
Soil Water Content (SWC) at t_1_ and t_2_ in the stress treatment at the reproductive stage. The dashed line indicates the starting SWC, at t_0_. In black are the control (CTRL) and in stripes the stressed (DS). Vertical bars represent standard deviation of means of the values taken on 4 plants.

Like the vegetative phase, there is a correlation with the SWC. In this case, at t_1_ the soil of Costoluto, Giallo, Quarantino and Pearson still contained an appreciable amount of water (Figure 14). Clearly at t_2_ the differences between CTRL and DS are amplified without an appreciable varietal difference; only Quarantino maintains a higher SWC than other stressed cultivars. Thus, once again the different perception of water scarcity likely corresponds to better management of the soil water resource in Quarantino.

**Figure 15:**
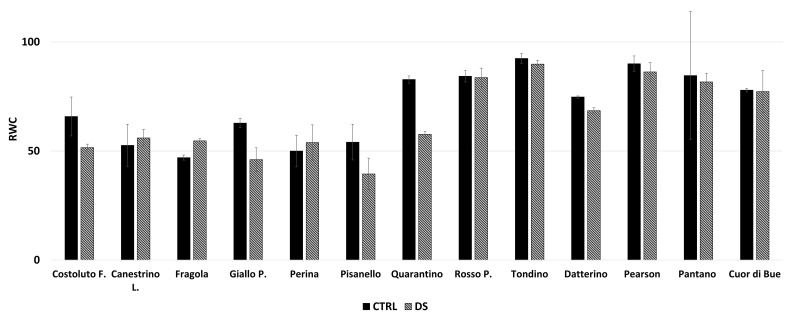
Relative Water Content (RWC) at t_2_ in the stress treatment at the reproductive stage. In black are the controls (CTRL) and in stripes the stressed (DS). Vertical bars represent standard deviation of means of the values taken on 3 leaves per plant.

The RWC was calculated for the aerial part of the plant. This parameter provides an interpretation of how water stress might affect plants differently [37]. Costoluto, Giallo, Pisanello, Quarantino and Datterino cultivars show a decrease in RWC compared to their own controls (Figure 15). RWC was established as an indicator of water status balance [38]. The decrease in RWC usually indicates a worse resistance to drought stress [39,40] and the cultivars maintaining RWC values comparable to their control are Canestrino, Fragola, Perina, Rosso, Tondino, Pearson, Pantano and Cuore di Bue.

As regards photosynthesis in the reproductive phase, the Quarantino cultivar has a value of A equal to 6.3 µmol m^−2^ s^−1^ at t_2_, a value like its own control (Figure S5). The cultivars Tondino, Perina, Pisanello, Costoluto and the commercial Pantano have a positive A greater than 2. However, in the commercial cultivar, the value at t_2_ differs particularly from its own control. The other cultivars have an A close to 0 showing that this parameter seems to be particularly affected by stress.

Once again, the WUE in the reproductive phase shows that the Quarantino maintains values comparable to control, indicating that it is not particularly affected by water stress (Figure S6). Other cultivars with a WUE value close to the control at t_2_ are Tondino, Pantano and Cuor di Bue. The cultivars Perina, Pisanello and the commercial Pearson also keep a comparable value. On the contrary, Giallo, Canestrino, Rosso, Costoluto, Datterino, and most of all Fragola are more sensitive to water stress as regards the WUE, as they have an extremely low value at t_2_. In general, there is an increase in WUE in all cultivars after a few days from the beginning of the stress (t_1_).

## 3. Discussion

The number and diversity of responses to drought define the ability of a plant species or cultivar to tolerate this abiotic stress [41]. Consequently, lower or higher susceptibility to drought is necessarily related to the plant genotype. Building on these facts, we screened tomato cultivars catalogued in the Regional Germplasm Bank of Tuscany and therefore adapted to the climatic and soil conditions of Tuscany. Plants were analyzed during both the vegetative and reproductive phases; behind that was the question of whether a given cultivar was specifically more tolerant in one phase than the other. This could disclose even more specific mechanisms of tolerance. To obtain the sought information, tomato plants were evaluated for a number of physio-morphological parameters that were subsequently integrated and correlated with each other.

In plants, the first perception of water deficit results in the closure of stomata, which leads to the decreasing of stomatal conductance. We found that the g_s_ of tomato plants is lower in stressed samples than in the corresponding control, suggesting that drought-stressed plants strongly perceive stress and consequently adapt [30,39]. Nevertheless, not all tomato cultivars behave the same way. Just to briefly summarize, in the vegetative phase the local cultivars Costoluto Fiorentino, Giallo di Pitigliano, Rosso di Pitigliano and Pisanello as well as the commercial Datterino show g_s_ close to zero at mid-stress. On the contrary, the cultivars Perina, Fragola, Tondino, Quarantino and the commercial Cuor di Bue are more tolerant, showing a non-varying conductance in the middle and final phase of stress. In the reproductive phase, the situation differs partially because the cultivars Perina, Giallo, Fragola, Canestrino, Rosso and the commercial Datterino, Pearson and Cuor di bue have a g_s_ close to zero at the mid time. The cultivar Quarantino also achieves to maintain an adequate conductance as well as the cultivars Costoluto, Pisanello, Tondino and the commercial Pantano.

Photosynthesis is another physiological target of primary importance for drought [4,30,31]. In the vegetative phase, Tondino, Quarantino, Fragola, Perina and Cuor di Bue show an A value different from zero, while photosynthetic activity is strongly affected at mid-stress in the other cultivars. This suggests that the five cultivars mentioned above are the most tolerant. However, distinctions are present in the reproductive phase because Canestrino, Fragola, Giallo, Rosso, Cuore di Bue, Datterino and Pearson show an A value close to 0, thus a strongly reduced photosynthesis. In contrast, the other cultivars have a positive A; since the A value of Quarantino at t_2_ is like the control, this is another indication of its higher drought tolerance. Because there are no studies on the same cultivars in the literature, we can refer to the work of Zhou [39], in which the tomato cultivar Arvento showed an A value different from 0 already at the first-time interval of combined stress (heat and drought) and was the most drought-tolerant cultivar. 

In this study, as observed by Mishra [42], none genotype showed differences in photosynthetic efficiency (Fv/Fm and PI) between stressed and control plants after eight days of stress. In an earlier study on Tuscan tomato cultivars under drought conditions, Conti [28] found that photosynthetic efficiency decreased from the fourteenth day of stress. Indeed, a brief period of drought usually does not affect the Fv/Fm parameter [10,42]. This is because the first response to drought (i.e., stomata closure) does not affect the ability of PSII to reduce the first electron transporter, Qa. In fact, the water-water cycle and photorespiration initially allow stressed plants to accomplish electron transport in a way comparable to control plants, avoiding photodamage to PSII [10]. In contrast, PI is a more drought-sensitive parameter than Fv/Fm [43]. In all stressed genotypes (except Perina, Rosso di Pitigliano, and Tondino Liscio), PI decreased significantly, differing from control values after 16 days of stress in the vegetative stage. In the reproductive phase, PI values show the same course as Fv/Fm. The cultivars Costoluto, Canestrino, Fragola and the commercial cultivar Datterino show a decline of PI already at t_1_ with a stronger reduction at t_2_. The cultivars Giallo and Quarantino differ from the other cultivars when their performance is compared to the control. On the contrary, the cultivars Perina, Pisanello, Rosso, Tondino and the commercial Pearson and Pantano have a PI that markedly decreases after t_1_.

At the vegetative stage, all photosynthetic parameters indicate Perina and Cuor di Bue (followed by Fragola, Quarantino, and Tondino) as the cultivars capable of maintaining photosynthetic activity. The reduction of A value in these cultivars is less significant than in the others and does not correspond to an irreversible damage of photosystems. On the contrary, the photosynthetic system is more compromised in the cultivars Pisanello, Canestrino, Giallo, and commercial Datterino. In the reproductive phase the situation is slightly different. It is straightforward to establish that the most tolerant cultivar is Quarantino because it shows excellent values for all the photosynthetic parameters. It is also equally simple to recognize the most susceptible cultivar, i.e., Fragola, because all photosynthetic parameters are negative or quite different from the control. The classification of other cultivars, such as Perina, on the base of the photosynthetic parameters is more complicated since in the stressed plants they indicate both better or worse condition compared to control.

Plant growth is clearly linked to photosynthesis as the decrease in photosynthesis rate leads to reduced biosynthesis of carbohydrates that are used for growth [44]. In all tomato cultivars at the vegetative phase, a sharp decrease in growth was observed after eight days of stress (GI_(1,0)_), except for Perina, Canestrino, Quarantino and Cuor di Bue. Significant differences have been found for the commercial Pantano and the cultivars Costoluto, Tondino, Giallo and Pisanello (Figure S7a). For the GI_(2,0)_, the growth index at the end of stress, a significant decrease was shown for all cultivars except for Quarantino, that is still comparable to its own control (Figure S7b). An earlier work of our on a subset of the tomato cultivars showed a difference in growth only after 16 days of stress [28]. In that case, however, the study was carried out in a growth chamber under controlled conditions while in this study plants were grown under natural-like conditions, especially in terms of temperature. We believe this might affect the time plants perceive water deficit. However, the cultivars whose growth was mostly affected by stress correspond when comparing this study to the earlier one. In the reproductive phase at the middle of stress, the GI_(1,0)_ does not show relevant data and values of most stressed cultivars are similar to their own control, except for Pisanello, Giallo and commercial cultivar Pantano, that show a significant decrease in growth (Figure S8a). At the end of the stress (GI_(2,0)_) drought significantly affected plant growth. In particular, the cultivars Costoluto, Pisanello, Tondino, Cuor di Bue, Datterino and Pantano suffered the most, with a marked difference in growth between control and stressed plants. On the other hand, the Canestrino, Fragola, Giallo, Perina, Quarantino, Rosso and Pearson cultivars showed a slighter difference in growth, but also high standard deviations like all other cultivars, thus data are difficult to interpret (Figure S8b). However, in general, plant growth is not particularly affected by cultivar type or stress condition because all data decrease in stressed plants compared to controls.

The WUE parameter (A/g_s_) expresses the photosynthetic capacity of plants to produce biomass per unit of water consumed [30] and is considered a useful parameter for evaluating the best performing plants under water deficit conditions [32]. In the vegetative phase, Perina and Fragola maintain a high WUE during the stress period. On the contrary, Pisanello shows an extremely low value of WUE already at mid-term stress. In the reproductive phase, Quarantino shows a high WUE value even at t_2_, indicating it as the most tolerant cultivar during this growth period. An adequate WUE value is also achieved by the cultivars Tondino, Perina, Pisanello and by the commercial Cuor di Bue, Pantano and Pearson. However, WUE increases in all cultivars during the first days of water deficit and then gradually decreases. Similar responses (i.e., increase of WUE in the first days of stress) were found for grapevine [45], potatoes [46], where a rapid decrease in WUE occurred at the end of stress, and for tomato cultivars in the Mediterranean area of study [30].

The increase in WUE under moderate drought conditions, such as those in the first days of stress, is due to the slow relative decrease of A in comparison to g_s_, which decreases more rapidly; for simplicity, we can assume a higher permeability of plants to incoming CO_2_ rather than outgoing H_2_O. One approach to increase WUE is changing the stomatal density: indeed, decrease in stomatal density triggers lower levels of g_s_ in drought-stressed plants with the same photosynthetic activity [33]. In our work, the Pisanello cultivar shows a higher density of stomata when subjected to drought, confirming a higher susceptibility to stress. Exactly the opposite case occurs for Perina, which has a lower stomatal density under stress, implying an adaptation to water deficit. The stomatal density of Quarantino and Fragola is unchanged between control and stressed plants, indicating less susceptibility to stress. 

By combining all data, we can discriminate the nine local cultivars into those most susceptible to drought and those most tolerant. We assume that the difference between susceptible and tolerant cultivars is because of drought tolerant cultivars having more efficient and protective mechanisms [20,47]. The data also allowed to differentiate cultivars on the basis of vegetative and reproductive stages. We used the tool PCA to identify tolerant and susceptible genotypes; PCA has already proved to be useful in many other studies [41,48,49]. Analysis by PCA and the correlogram data-derived dendrogram confirmed the classification of cultivars into two groups (one tolerant and the other susceptible) at the level of vegetative stage. The cultivars Perina and Fragola are those that perform better to drought stress and can therefore be recognized as the most tolerant; on the other hand, the cultivar Pisanello is the most susceptible to drought, while the cultivar Quarantino shows an intermediate behavior. 

At the reproductive stage, the situation is different. The first PCA revealed that drought affects and distinguishes controls from stressed plants. The second PCA differentiates two groups, and the detailed analysis of all parameters indicates that Quarantino is the most tolerant cultivar, while Fragola is the most susceptible. Clustering does not reflect the groups obtained by PCA. We hypothesize that cultivars at the reproductive growth stage do not exhibit well-standardized behavior. Because clustering was done by referring to plant behavior during the entire stress period and not just at t_2_, this affected the distinction into groups. In the reproductive phase, distinction between genotypes occurs just at the end of stress. For this reason, the cluster division obtained by PCA at t_2_ is more relevant than the parameter-based clustering during the entire stress period.

## 4. Materials and Methods

### 4.1. Plant Material

Seeds of nine Tuscan tomato cultivars, namely ‘Costoluto Fiorentino’, ‘Canestrino di Lucca’, ‘Fragola’, ‘Rosso di Pitigliano’, ‘Giallo di Pitigliano’, ‘Pisanello’, ‘Quarantino ecotipo Valdarno’, Tondino Liscio da Serbo Toscano’ and ‘Perina a Punta della Valtiberina’, were obtained from the Regional Germplasm Bank of Tuscany (Tuscany, Italy). No permissions were necessary to collect seeds. The Regional Germplasm Bank of Tuscany undertook the formal identification of samples. Four commercial cultivars, namely ‘Cuore di Bue’, ‘Datterino’, ‘Pantano’ and ‘Pearson’, have been chosen among many other commercial cultivars because of their wide commercialization all over Italy; the corresponding seeds were provided by local retailers.

### 4.2. Growth Conditions and Stress Treatment

Seeds were germinated in Petri dishes on filter paper soaked with distilled water at a constant temperature of 25 °C in the dark. Afterwards, seedlings were transferred to a greenhouse (Botanical Garden, University of Siena) and planted in a tray with wells (4 × 5 × 6 cm) at 25 °C. For each cultivar, 10 plants were studied during the vegetative phase and 8 plants during the reproductive growth phase. For studies at the vegetative phase, plants were transferred into square PE pots (15 cm side, 20 cm height), while for studies at the reproductive stage PE pots had an upper diameter of 28 cm, a base diameter of 22 cm, and a height of 24 cm. The substrate used for repotting operations was the VIGOR PLANT^®^ RADICOM BIO. For each cultivar and growth phase, half of plants were used as control (CTRL) and the other half were subjected to drought stress (DS). Until the beginning of water deficit treatment, all plants were well-watered. For studies at the vegetative phase, the drought treatment began when plants were 30/40 cm high, corresponding to 45 d after germination; the stress condition was maintained for 16 d and consisted in complete watering withdrawal; the CTRL group was kept in fully irrigated regime for the whole period.

For studies at the reproductive phase, the drought treatment began when plants were flowering, and the first fruits started to grow. Plants were around 120 cm high at the beginning of stress, the drought treatment lasted for 20 d and consisted in complete watering withdrawal; the CTRL group was kept in fully irrigated regime for the whole period. 

The timing of the drought was chosen following Landi [50], Sànchez-Rodrìguez [51], Nuruddin [27] and our previous work in a growth chamber [28]. The experimental period was divided into 3 time points for each phase: time point 0 (t_0_) corresponds to the beginning of stress; time point 1 (t_1_) is the intermediate stage of stress; time point 2 (t_2_) is the end of stress. Plants in the reproductive phase were also subjected to a recovery step, consisting of full reirrigation of drought-stressed plants after t_2_ for two weeks (recovery time point, RW). At each time point, all required parameters were taken, and all samples were harvested, immediately put in liquid nitrogen, and stored at −80 °C until use.

### 4.3. Temperature and Relative Humidity

Each phase was performed during July in a greenhouse with a complete randomized scheme. The greenhouse facility prevented accidental wetting of plants but allowed solar illumination, temperature, and humidity parameters to be comparable to those outside. However, temperature and humidity values were collected hourly by an EBI 20-TH1 (ebro^®^) datalogger, daily mean and standard deviation computed separately for day and night hours. During the vegetative phase, the mean temperature and humidity in daytime hours were 34.7 ± 2.6 °C and 46.8 ± 6.2%, respectively; during nighttime hours, the mean temperature was 25.3 ± 1.7 °C while the mean humidity was 60.9 ± 6.3%. During the reproductive phase, an average temperature of 32.7 ± 3.8 °C and humidity of 50.7 ± 8.4% was recorded during daytime hours, while temperature and humidity were 23.9 ± 2.1 °C and 64.7 ± 3.2%, respectively, during nighttime hours. The values were very close to those recorded in Siena in July.

### 4.4. Soil Water Content

The Soil Water Content (θ_g_) was evaluated for each pot. Soil samples were weighted (m_wet_), put over-night in an oven at 105° C and then weighted again (m_dry_). Soil water content was calculated as:

(1) $$\theta_{g}=\frac{m_{water}}{m_{soil}}=\frac{m_{wet}-m_{dry}}{m_{dry}},$$

where

- θg = Gravimetric Water Content,
- mwater = mass of water contained in the samples,
- msoil = sample soil mass,
- mwet = wet soil sample mass,
- mdry = dry soil sample mass.

The mean and standard deviation for each cultivar and phase was calculated at t_0_, t_1_ and t_2_.

### 4.5. Relative Water Content

The leaf relative water content (RWC) was determined as follows [52,53]. Completely expanded and mature leaves at t_2_ were cut, leaving a petiole of about 1 cm, immediately inserted into plastic bags with the petiole down, closed and stored in the dark. Each leaf was weighed with their own plastic bag (TFW-Total Fresh Weight) using a Gibertini-EUROPE_500 balance. Then, 2–3 mL of CaCl2 were added. Samples were incubated for 8 h, allowing them to absorb the CaCl2 solution. Subsequently, leaves were removed from the plastic bag and placed between two paper towels to absorb the excess water. To determine the turgid weight (TW-Turgid Weight), each leaf was weighed. Then, leaves were placed into a paper bag and heated in oven at 60 °C for 3–4 d. Finally, samples were weighed to determine the dry weight (DW-Dry Weight). The RWC of leaves was calculated as:

(2) $$RWC=\frac{\left(TFW-BW\right)-DW}{TW-DW}\times 100,$$

where

- RWC = Relative Water Content,
- TFW = Total Fresh Weight,
- BW = Bag Weight,
- DW = Dry Weight,
- TW = Turgid Weight.

The mean and standard deviation for each cultivar were calculated.

### 4.6. Growth Index

The growth index (GI) was calculated as:

(3) $$GI_{f,i}=\frac{h_{f}-h_{i}}{2},$$

where

- hf = final height,
- hi = initial height.

Heights were measured at t_0_, t_1_ and t_2_ for both vegetative and reproductive phases. The height of each plant was measured with a meter stick parallel to the stem, from the base up to the highest internode. Three GIs were calculated for each plant: GI_1.0_ indicates the growth between t_0_ and t_1_, GI_2.1_ between t_1_ and t_2_, while the total growth is expressed by GI_2.0_. The mean and standard deviation of GI for each time-point, cultivar and growth phase were computed.

### 4.7. Stem Diameter

The stem diameter was measured with a digital caliber (POWERFIX^®^, Neckarsulm, Germany) at t_0_, t_1_ and t_2_. The diameter was measured about 7 cm from the base of stems, which was marked during the first measurement. The mean and standard deviation for each plant and growth phase were computed.

### 4.8. Efficiency of Photosynthesis

Photosynthetic efficiency was evaluated by using a fluorometer Handy PEA 2000 (Hansatech Instruments King’s Lynn, Norfolk, UK) analyzing Fv/Fm and the performance index (PI). The parameter Fv/Fm indicates the maximum quantum efficiency of Photosystem II., where Fv is the difference between the maximum fluorescence signal (Fm) and the basic fluorescence. The parameter PI shows variations of the entire photosynthetic apparatus, including photosystem I (PSI) and II (PSII). For each growth phase and cultivar, Fv/Fm and PI were collected at t_0_, t_1_ and t_2_. Finally, the mean and standard deviation were calculated.

### 4.9. Leaf Gas Exchange: Stomatal Conductance and Photosynthesis

The LI-6400XT Portable Photosynthesis System (LI-COR Inc., Lincoln, NE, USA) equipped with 6400-40 Leaf Chamber Fluorometer were used to analyze CO_2_ and H_2_O gas exchange, and intercellular concentration of CO_2_ (Ci), stomatal conductance (g_s_), and net photosynthesis (A) were calculated. Inside the chamber, the relative humidity (30/70) and the temperature (set to 30 °C) were measured. The light in chamber, the CO_2_ concentration was maintained at 400 µmol mol^−1^, the relative humidity at 40 to 50%, temperature at 30 °C and the PAR was set to 1500 μmol s^−1^ (values close to the average growth conditions in the greenhouse). The first fully expanded leaves from the apex of plants were used for measurements. The measurements of each plant and phase were carried out four times: at t_0_, t_0-1_ (between t_0_ and t_1_) t_1_, and at t_2_ for the vegetative phase; at t_0_, t_1_, t_2_ and t_R_ for the reproductive phase. Finally, the mean and standard deviation were computed. The A/g_s_ ratio, which expresses the water use efficiency (WUE), was calculated for all cultivars.

### 4.10. Morphometric Evaluation of Leaf

In the vegetative phase, at each time-point (t_0_, t_1_ e t_2_) and for selected cultivars, pictures of three leaves per plant at the same developmental stage were taken. Pictures were examined with the software ImageJ (National Institute of Health, Bethesda, MD, USA) to determine:

- Leaf Area (LA),
- Lamina Length (LaL),
- Lamina Width (LaW) (for this parameter 3 measures were taken for each leaf).

Finally, the mean and standard deviation were computed.

### 4.11. Stomatal Density

The stomatal density was calculated at t_2_ during the vegetative phase of selected cultivars. Three leaves from each plant were sampled at the same developmental stage. On the lower surface of leaves, a thin layer of transparent nail polish was uniformly applied according to Xu and Zhou [54]. Once dried, the nail polish was pulled away and the molds obtained were put onto a microscope slide. Samples were examined with the optical microscope Zeiss Axiophot (Oberkochen, Germany). For each mold, 10 pictures were taken, and stomata were counted using ImageJ. Stomata number per leaf area (mm^2^) expresses stomata density. Finally, the mean and standard deviation were calculated.

### 4.12. Statistical Analysis

Principal Component Analysis (PCA), correlograms and the dendrogram were performed with RStudio IDE (RStudio PBC, Boston, MA, USA). In particular, the corrplot package was used for the analysis of correlation coefficients and their visualization. Raw data were normalized then KMO adequacy and Bartlett’s test were performed before factor analysis while orthogonal varimax rotation method was chosen for PCA. Clustering was performed by UPGMA hierarchical cluster analysis on the base of Mahalanobis distance metric.

## 5. Conclusions

We have performed a detailed analysis of several physiological and morphological parameters, which highlighted critical differences of Tuscan tomato cultivars in the responses to drought. This allowed to classify the cultivars based on their tolerance ability.

Local cultivars exhibit a genotype-dependent response to drought more than commercial cultivars, in both vegetative and reproductive phases of growth. We therefore distinguished a different behavior for all nine local and four commercial cultivars. Two groups of plants were recognized: one composed of the cultivars that are more tolerant to drought, the other one of plants that are more susceptible. For the vegetative phase, the most tolerant cultivar is Perina while in the reproductive phase Quarantino performs better. This indicates that the relationship between plants and water deficit also depends on the individual growth phase. Perina and Quarantino are the cultivars that behave intermediately (i.e., medium tolerance) in the reproductive and vegetative phase, respectively.

From a more general point of view, this confirms that biodiversity is a great reservoir from which to retrieve crucial genetic traits, both in terms of productivity and tolerance to abiotic stresses. In the future, the most drought-tolerant tomato cultivars could be chosen for breeding programs, also according to their productivity. Another beneficial point of using drought-tolerant plants is that sustainable agriculture benefits from drought-tolerant cultivars because, when used in combination with appropriate irrigation plans, they can improve agrobiodiversity and save significant amounts of irrigation water.

## Data Availability

Data available on request due to restriction.

## Supplementary Materials

The following are available online at https://www.mdpi.com/article/10.3390/plants10091826/s1, Figure S1: Trend of stomatal conductance (g_s_) for the vegetative phase. The black straight line indicates the control trend (CTRL) while the dashed line the stress trend (DS). Vertical bars represent standard deviation of averages of the values taken on five plants. Figure S2: Course of photosynthesis (A) for the vegetative phase. The black straight line indicates the control trend (CTRL) while the dashed line the stress trend (DS). Vertical bars represent standard deviation of averages of the values taken on five plants. Figure S3: Water Use Efficiency (WUE) trend for the vegetative phase. The black straight line indicates the control trend (CTRL) while the dashed line the stress trend (DS). Vertical bars represent standard deviation of averages of the values taken on five plants. Figure S4: Trend of stomatal conductance (g_s_) for the reproductive phase. The black straight line indicates the control trend (CTRL) while the dashed line the stress trend (DS). Vertical bars represent standard deviation of averages of the values taken on four plants. Figure S5: Course of photosynthesis (A) for the reproductive phase. The black straight line indicates the control trend (CTRL) while the dashed line the stress trend (DS). Vertical bars represent standard deviation of averages of the values taken on four plants. Figure S6: Water Use Efficiency (WUE) trend for the reproductive phase. The black straight line indicates the control trend (CTRL) while the dashed line the stress trend (DS). Vertical bars represent standard deviation of averages of the values taken on four plants, Figure S7: Growth Index (GI) for the vegetative phase. Controls (CTRL) are in black while stressed (DS) samples are in stripes. Error bars represent standard deviation of means of values taken on four plants. (**a**) The GI_(1,0)_ indicates the growth between t_0_ and t_1_. (**b**) The GI_(2,0)_ indicates the growth between t_1_ and t_2_. Figure S8: Growth Index (GI) for the reproductive phase. Black bars are the control (CTRL) while striped bars are the stressed (DS) samples. Error bars represent standard deviation of means of values taken on four plants. (**a**) The GI_(1,0)_ indicates the growth between t_0_ and t_1_. (**b**) The GI_(2,0)_ indicates the growth between t_1_ and t_2_.
