# Supplementary figures and images for: Morpho-Physiological Classification of Italian Tomato Cultivars (Solanum lycopersicum L.) According to Drought Tolerance during Vegetative and Reproductive Growth
Source: Plants (Basel). 2021 Sep 2;10(9):1826. doi: 10.3390/plants10091826 (PMC8468351; doi:10.3390/plants10091826)

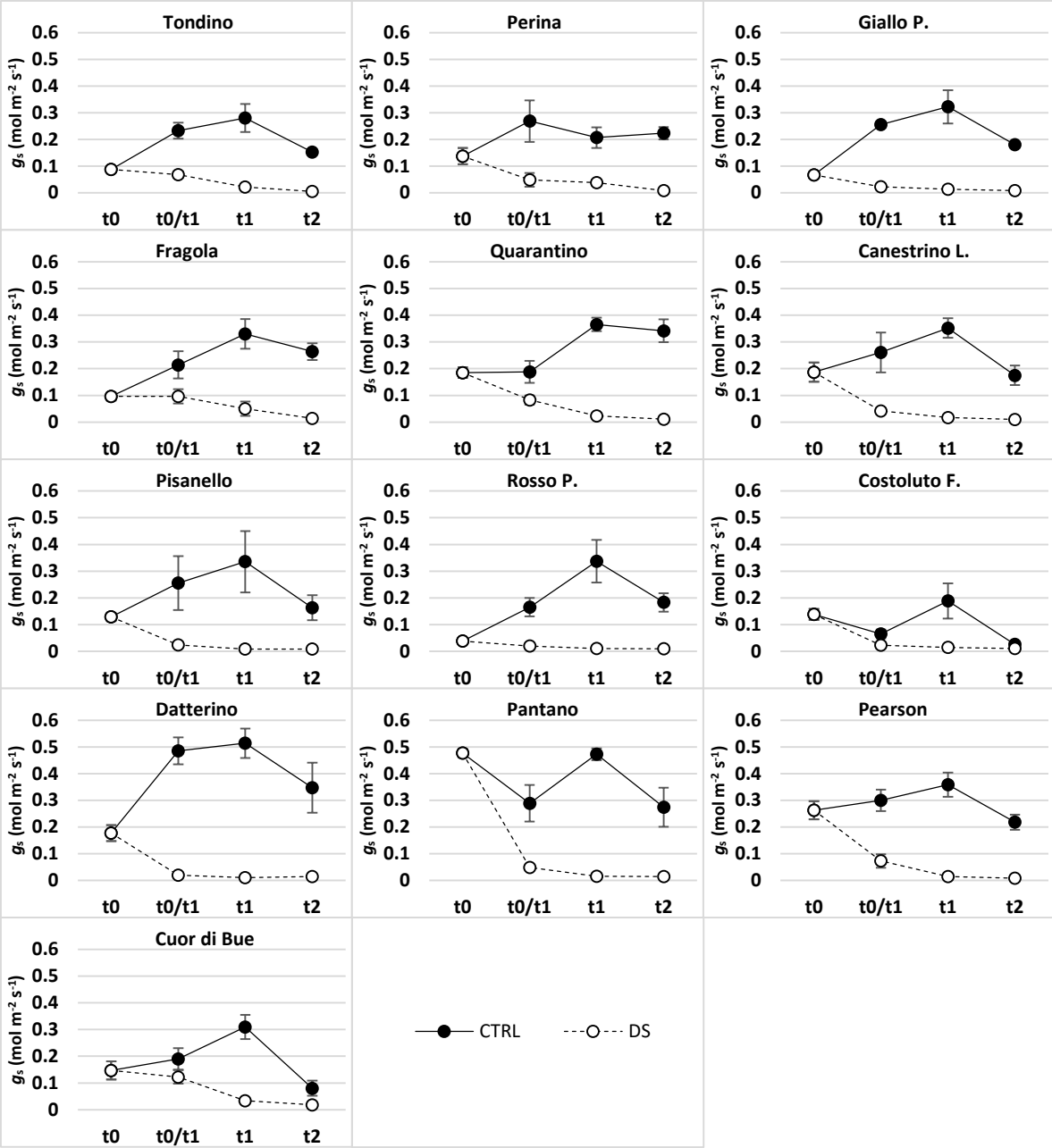

Supplement: Supplementary file 1 [file plants-10-01826-s001.zip › Figure S1.pdf]

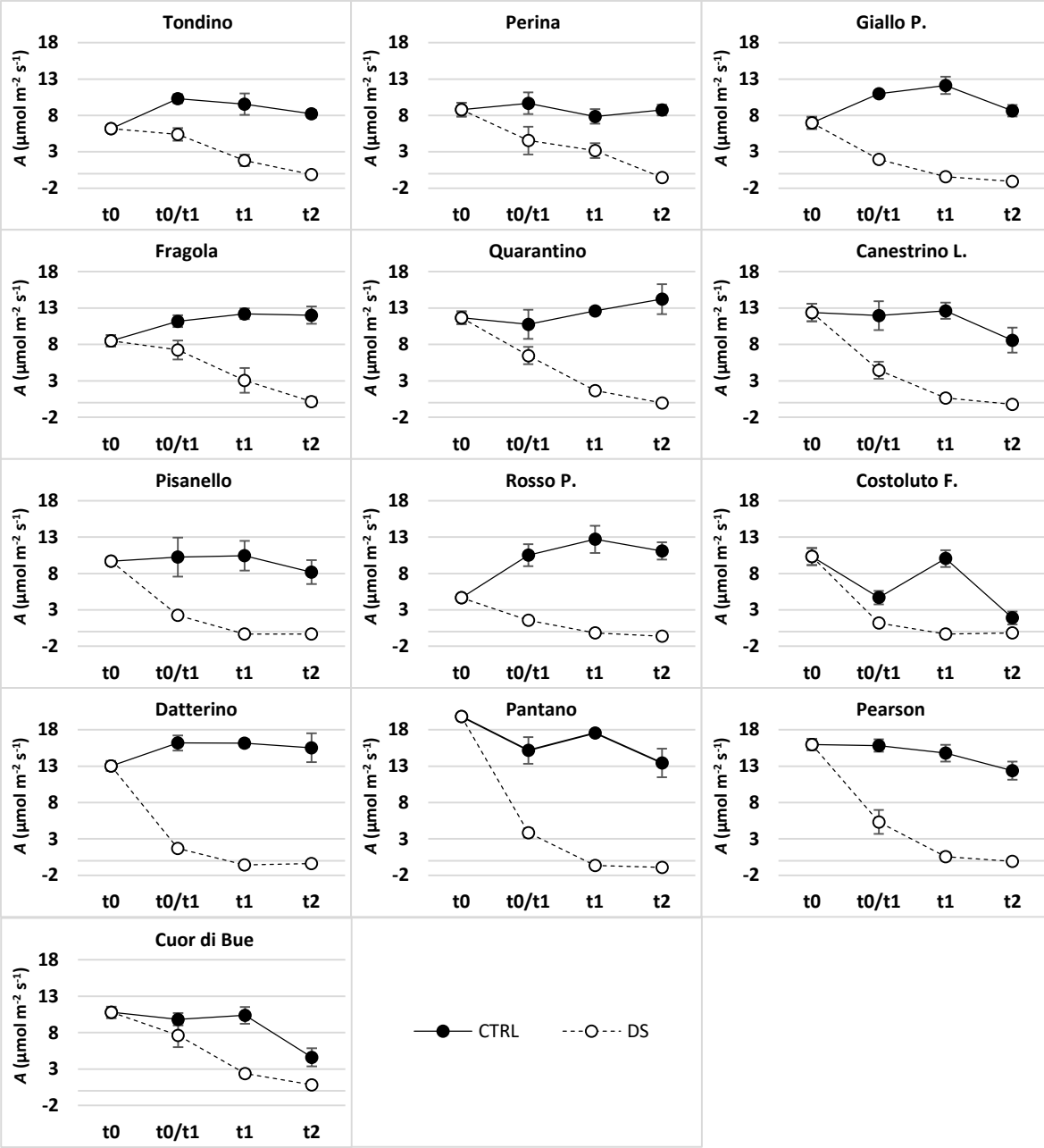

Supplement: Supplementary file 1 [file plants-10-01826-s001.zip › Figure S2.pdf]

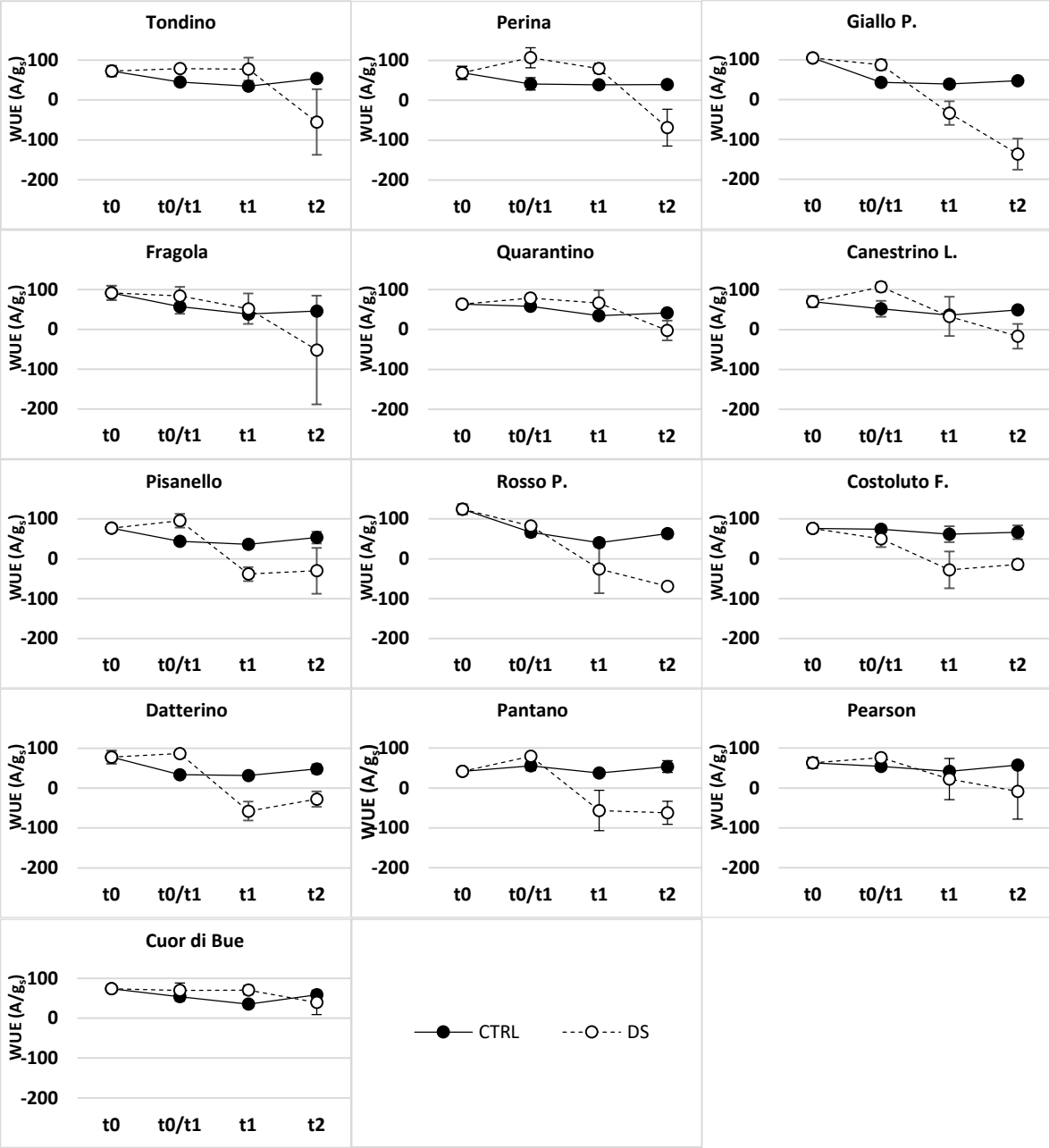

Supplement: Supplementary file 1 [file plants-10-01826-s001.zip › Figure S3.pdf]

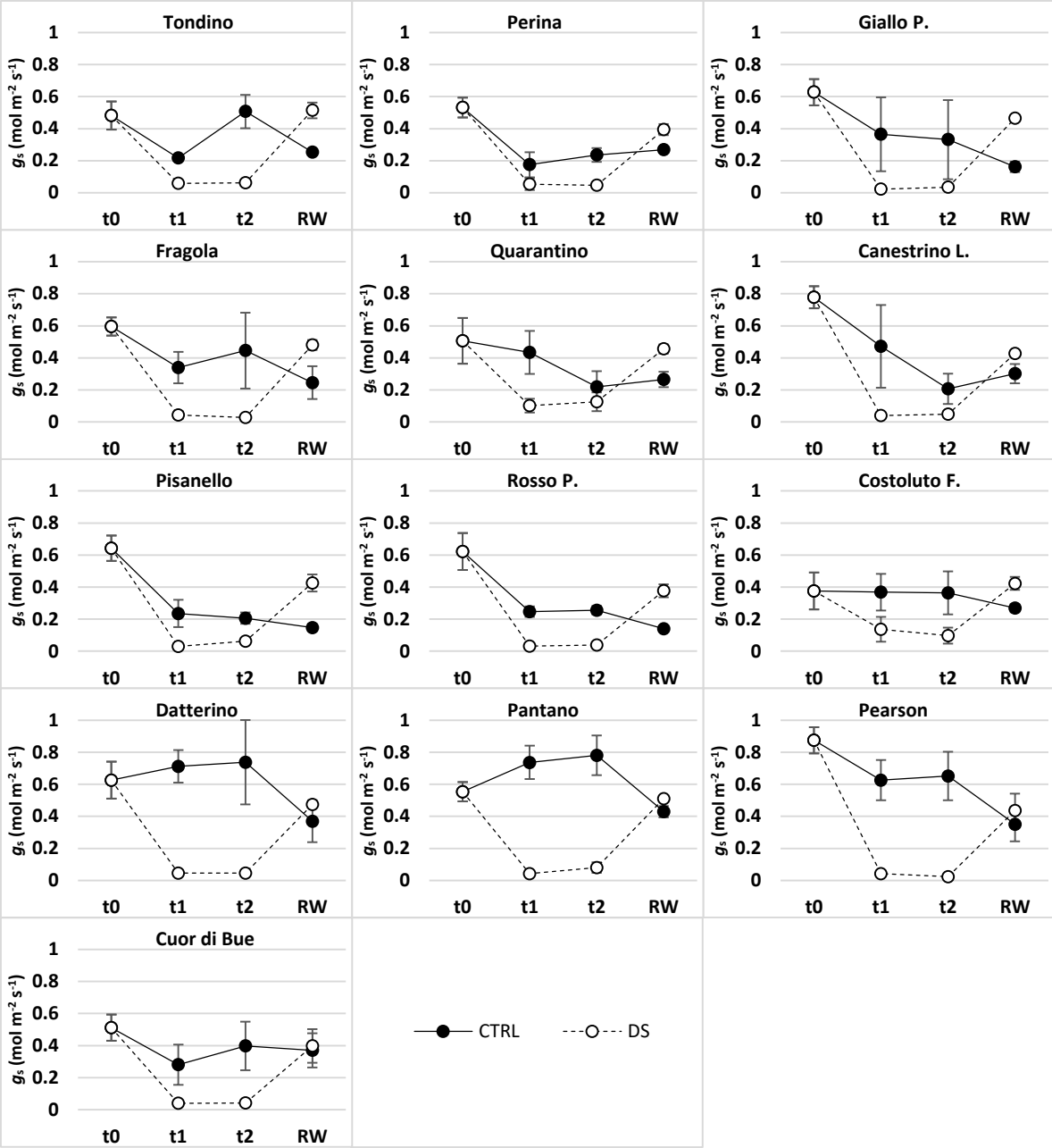

Supplement: Supplementary file 1 [file plants-10-01826-s001.zip › Figure S4.pdf]

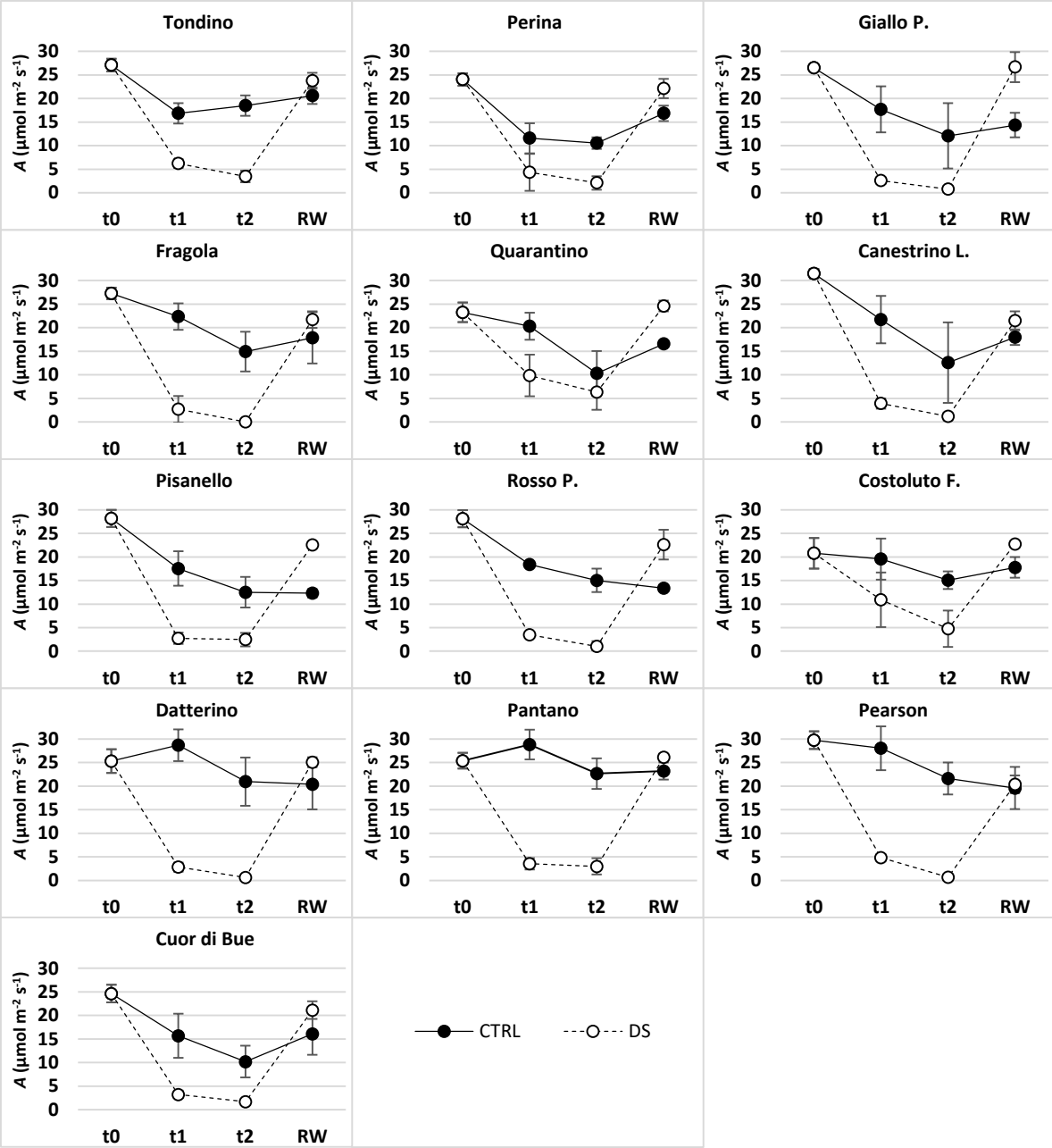

Supplement: Supplementary file 1 [file plants-10-01826-s001.zip › Figure S5.pdf]

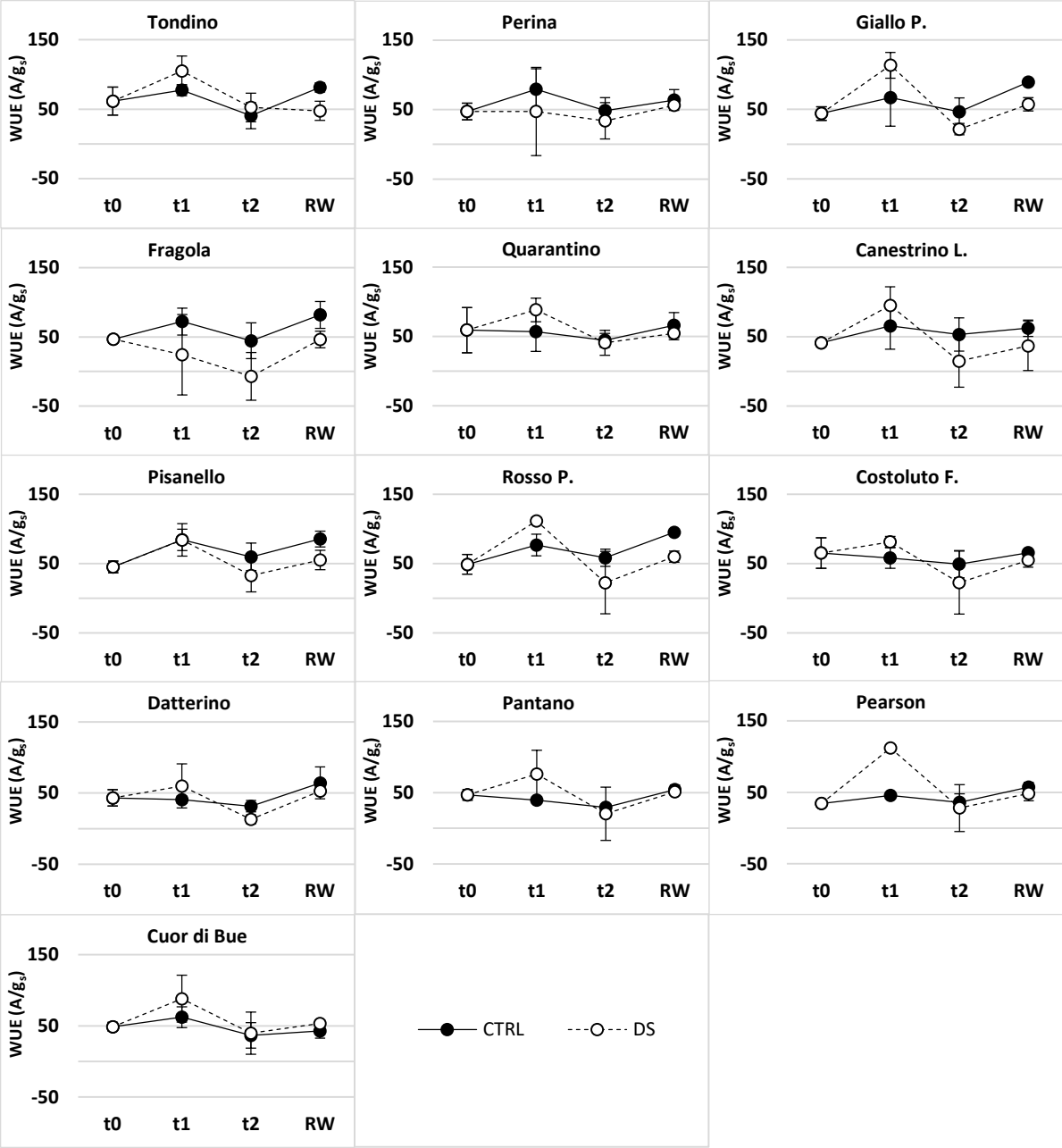

Supplement: Supplementary file 1 [file plants-10-01826-s001.zip › Figure S6.pdf]

a. 8

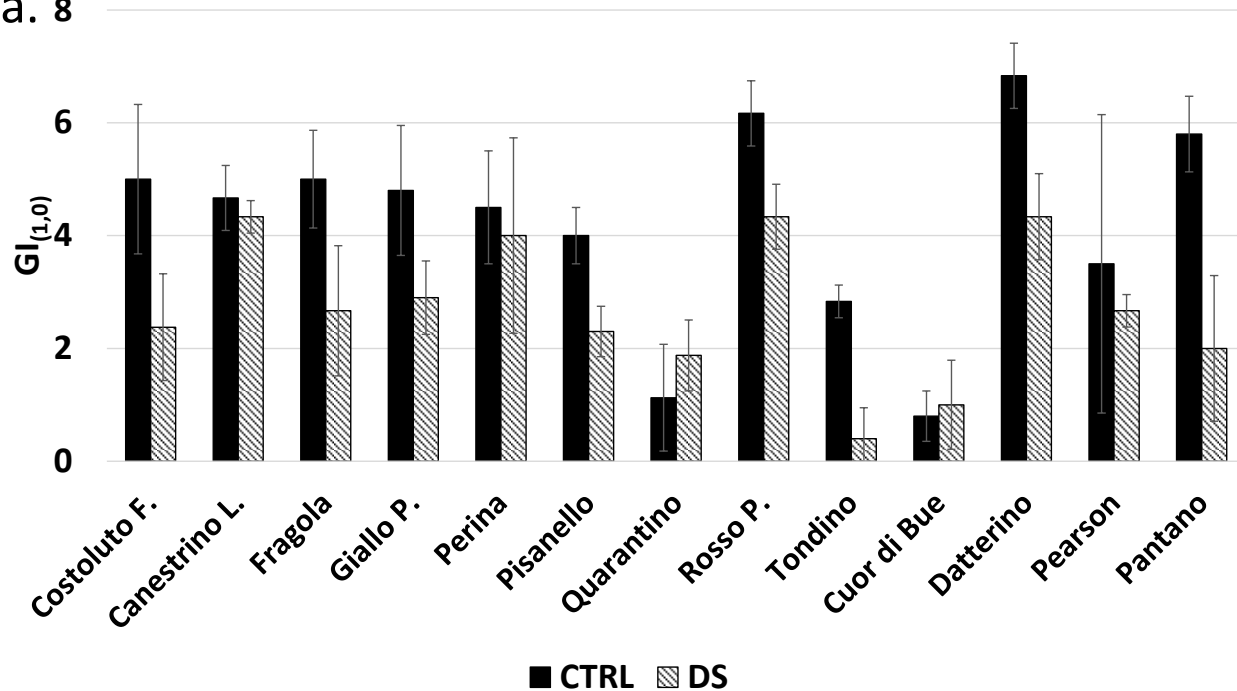

b. 12

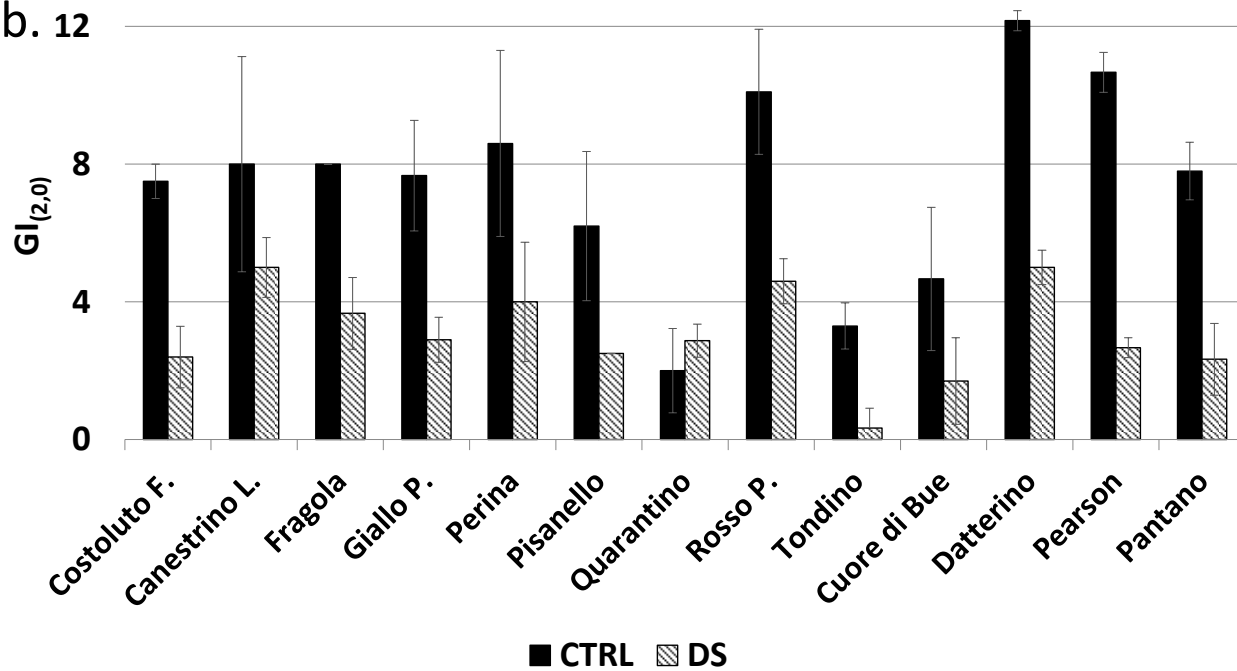

Supplement: Supplementary file 1 [file plants-10-01826-s001.zip › Figure S7.pdf]

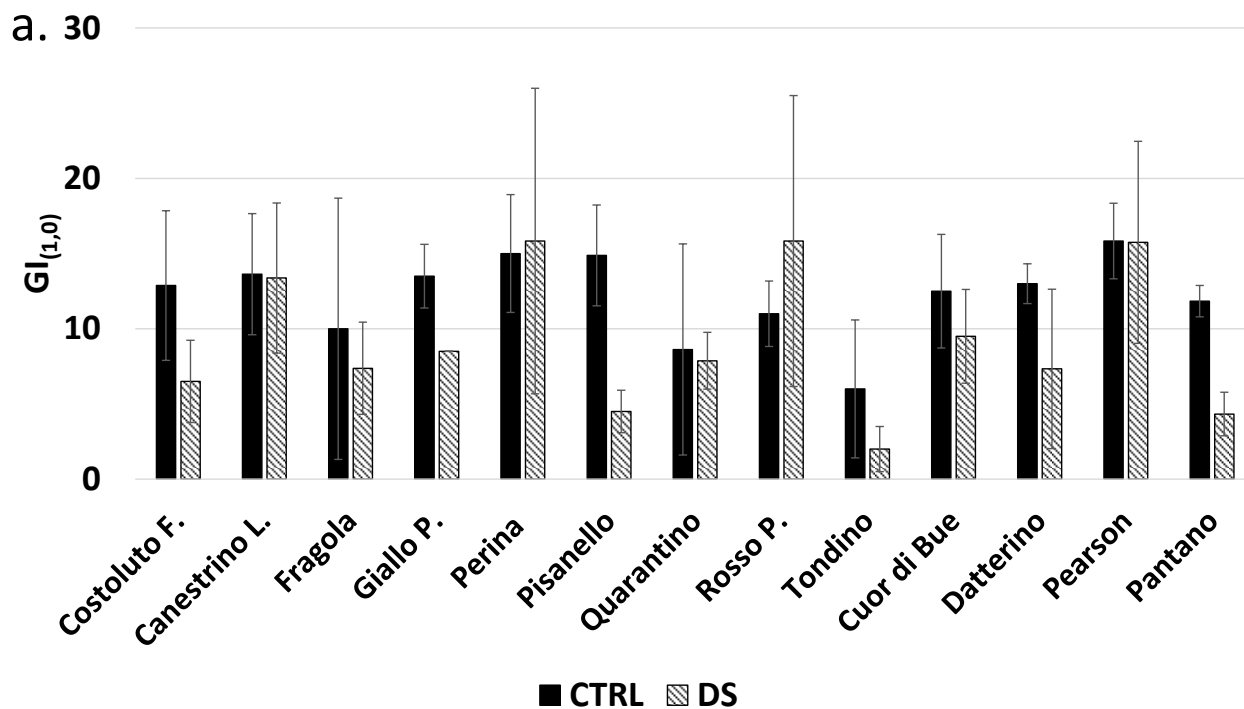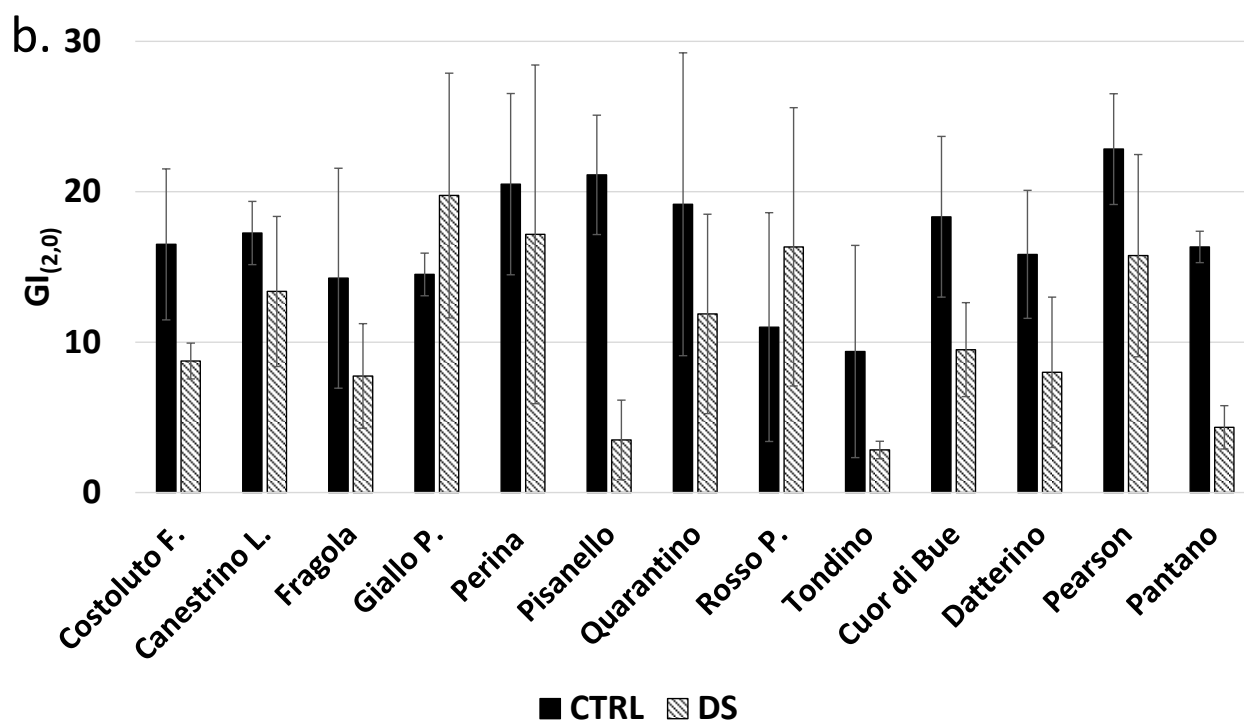

Supplement: Supplementary file 1 [file plants-10-01826-s001.zip › Figure S8.pdf]
